# Primary Pneumatosis Intestinalis of Small Bowel: A Case of a Rare Disease

**DOI:** 10.1155/2014/350312

**Published:** 2014-11-17

**Authors:** Daniela Berritto, Raffaello Crincoli, Francesca Iacobellis, Francesca Iasiello, Nunzia Luisa Pizza, Francesco Lassandro, Lanfranco Musto, Roberto Grassi

**Affiliations:** ^1^Depatment of Radiology, Second University of Naples, P.za Miraglia 2, 80138 Napoli, Italy; ^2^Depatment of Radiology, Ospedale Landofi ASL, Solofra Indirizzo, Via Melito, 83029 Solofra, Italy; ^3^Depatment of Radiology, Azienda ospedaliera “V. MONALDI,” Via Leonardo Bianchi, 80131 Napoli, Italy; ^4^Depatment of Radiology, Ospedale Criscuoli. Via Quadrivio, Sant'Angelo dei Lombardi, 83054 Avellino, Italy

## Abstract

Pneumatosis intestinalis (PI) is a condition in which multiple gas-filled cysts are located in the bowel wall; it can represent a wide spectrum of diseases and a variety of underlying diagnoses. The present report describes the case of an 86-year-old man with symptomatic primary PI of small bowel treated with surgical approach after periodic episodes of cysts rupture and superimposed inflammation revealed on the basis of a clinical suspicion thanks to abdominal computed tomography. Moreover, after one year of followup, there has been no recurrence of digestive symptoms.

## 1. Introduction

Pneumatosis intestinalis (PI) is a finding characterized by the presence of gas within the bowel wall [1, 2]. Two main theories have been proposed in the medical literature. A mechanical theory hypothesizes that gas dissects into the bowel wall from either the intestinal lumen or the lungs via the mediastinum due to some mechanism causing increased pressure (i.e., bowel obstruction or emphysema). A bacterial theory proposes that gas-forming bacilli enter the submucosa through mucosal rents or increased mucosal permeability and produce gas within the bowel wall [3, 4].

PI has been classified as primary (idiopathic) and secondary. Primary PI (15%) is generally nonsymptomatic and diagnosis is often occasional. This form is usually located in the colon. Males are more often affected and the age group most affected is between the fourth and sixth decades of life [5].

Secondary PI (85%) has been associated with numerous coexisting disorders of the gastrointestinal tract or the respiratory system, such as chronic obstructive pulmonary disease, intestinal obstruction, ischemic bowel disease, necrotizing enterocolitis in premature infants, immunodeficiency such as AIDS, bacterial and/or viral infection, and drug therapy [6–13]. Morphologically PI may occur in two different types: bubble-like (cysts in intestinal wall), typical of the primary type, and band-like (continuous lines), correlated with secondary PI [14, 15].

We present a rare case of idiopathic bubble like (cystoides) PI of the small bowel.

## 2. Case Report

An 86-year-old man was admitted to the emergency department presenting with abdominal pain, subobstructive episode, and constipation alternately to diarrhea. His previous medical history revealed a cholecystectomy.

Abdominal examination at admission revealed a diffuse distention and mild rebound tenderness. Laboratory studies revealed unremarkable serum blood count, electrolytes, and liver biochemistry.

Abdominal computed tomography (CT) without intravenous contrast demonstrated some gas-filled cysts within the small bowel wall, not associated with free intraperitoneal air. Based on the CT finding of air within the bowel wall, the patient was diagnosed with small bowel PI. The patient was not treated and he was discharged at his own request.

**Figure 1:**
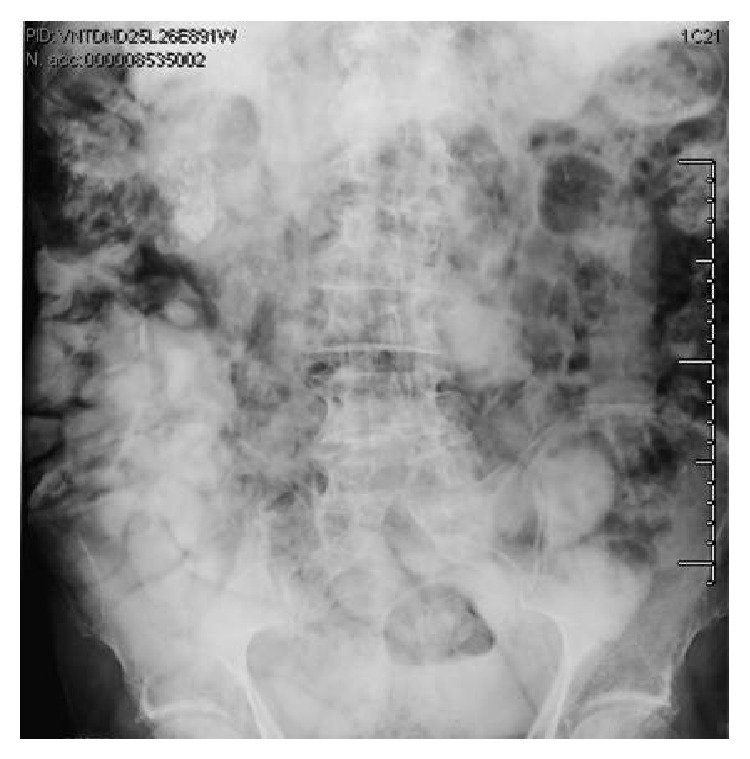
Plain abdominal film showing gas in the jejunal wall.

**Figure 2:**
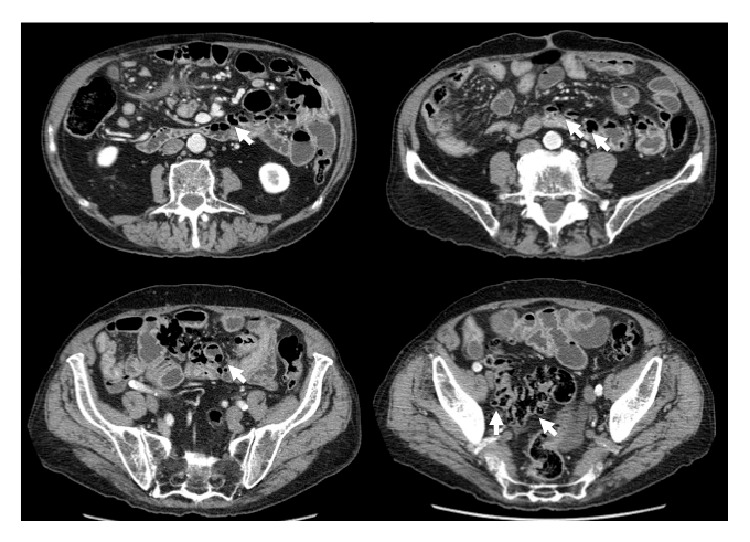
Computed tomography showing presence of gas in the intestinal wall (harrows).

One month later, presenting with a new episode of generalized abdominal pain, the patient underwent abdominal ultrasound (US) and plain abdominal film. The latter showed a large amount of intramural gas within some small bowel loops (Figure 1); US was useless due to intestinal tympanites. Abdominal CT with intravenous contrast administration was performed further to evaluate the pneumatosis and to investigate any possible complications. The scans confirmed the presence of small gas-filled cysts arising from the wall of the jejunum and ileum (Figure 2).

**Figure 3:**
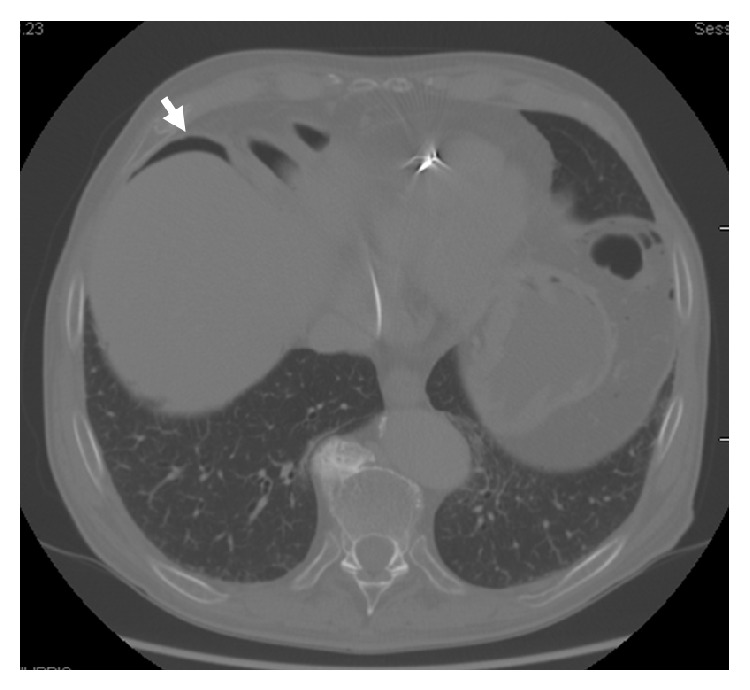
Abdominal CT scan showing free intraperitoneal air near the liver.

The onset of subobstructive symptoms over the following first month led the patient to repeat abdominal CT examination with intravenous contrast. It demonstrated the presence of small bowel PI with intraperitoneal free air near the liver and in the mesenteric fat due to the rupture of intramural gaseous cysts into the peritoneal cavity (Figure 3). A laparotomy was performed revealing some loops adherent to the parietal peritoneum due to earlier rupture of cysts with superimposed inflammation.

**Figure 4:**
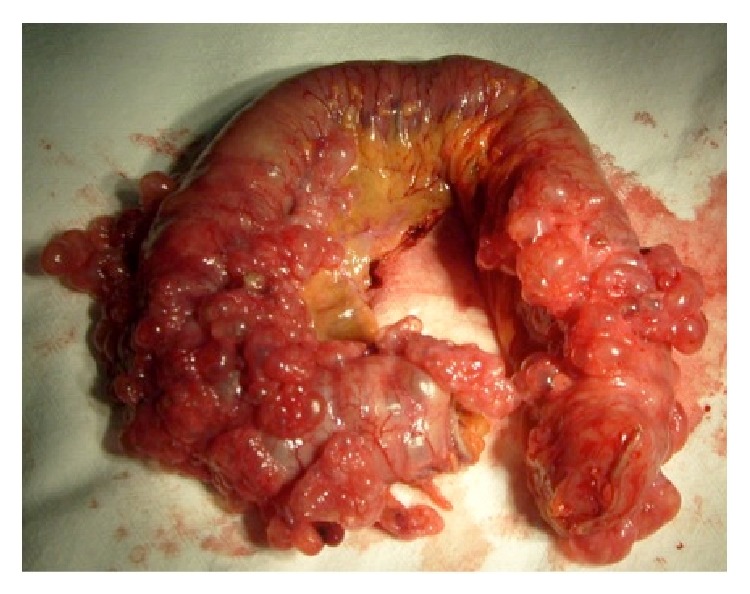
Gross appearance of the resected jejunum revealing multiple gas-filled cysts distributed within the bowel wall.

The most prominent affected segment of around 50 cm in length was resected (Figure 4) and lysis of adhesions was performed followed by jejunum resection with a side to side anastomosis.

Histological study revealed cysts located within the subserosa, with giant cells around the cystic wall.

After one year of followup, the patient was still asymptomatic.

## 3. Discussion

PI is radiologically characterized by cystic or linear collections of gas in the subserosa or submucosa of the gastrointestinal tract [4, 16]. It is an uncommon entity which recently obtained increased attention due to improved radiographic identification.

The first description of pneumatosis cystoides intestinalis was made by Du Vernoin as a postmortem observation [17].

In most cases, PI is an incidental finding, whereas in others PI is secondary to a wide variety of gastrointestinal and nongastrointestinal diseases [18, 19].

Clinically, PI ranges from benign disease, which does not require treatment, to more severe conditions needing oxygen, intravenous hydration, and antibiotics, to a life-threatening entity requiring immediate surgery.

In cases of PI due to benign causes, especially PI associated with pulmonary disease, the patients are usually asymptomatic and often only necessitate conservative therapy. Some patients may have mild abdominal discomfort, which is usually related to the underlying associated medical condition. Physical examination is rarely abnormal unless there are peritoneal signs from intestinal perforation in cases of PI due to life-threatening causes [3, 18, 20, 21].

In our case, the persistence of symptoms, due to inflammatory adhesions subsequent to episodes of asymptomatic rupture of the cysts, has made it necessary for lysis of adhesions. Since the bubbles were limited only to the small bowel, it was possible to perform a preventive partial jejunal and ileal resection in order to prevent further acute episodes.

Endoscopy, barium enema, US, plain abdominal film, and abdominal CT can be supportive in diagnosing PI.

In our case endoscopy was not performed due to the location of cysts into the small intestine, such as for barium enema. However, regarding the latter some authors have warned that PI could be confused with intestinal polyposis on barium enema, as they have very similar appearance [2, 22–26].

US can also be used to detect PI [27–29]. This technique is more commonly applied to the pediatric patient in whom avoidance of ionizing radiation is preferred but in most cases it is hampered by the presence of tympanites, as in our case. PI seen on US has been described as linear or focal echogenic areas within the bowel wall. It can also appear as a continuous echogenic ring in the bowel wall [29–31].

Plain abdominal film can be useful in identification of PI but it is more recommended for the study of complications, such as the pneumoperitoneum that is detectable on erect chest radiography by the presence of subdiaphragmatic air or by the evidence of air pockets on supine plain abdominal film [32].

CT has greater sensitivity in diagnosing PI than plain abdominal film or US since it can distinguish PI from intraluminal air or submucosal fat [18, 22–26].

The ability to study the bowel wall in the coronal, sagittal, and axial planes may allow a more confident diagnosis of PI and portal venous gas. Because CT is more sensitive than plain abdominal film in detecting PI, it can be used to clarify ambiguous radiographic findings and also to search for potential causes. On both plain abdominal film and CT, PI usually appears as a low-density linear or bubbly pattern of gas in the bowel wall. It can be a combination of both linear and bubbly bowel-wall gas. There also may be circular collections of gas in the bowel wall. CT has also been shown to be more sensitive than plain abdominal film in detection of some complications like portal and portomesenteric gas or the presence of pneumoperitoneum detected even with 1-2 mL of free intraperitoneal air [33].

In our case, CT allowed to identify complications consisting of intraperitoneal free air near the liver and in the mesenteric fat, without signs of portomesenteric air. On CT scans we recognized a typical primary cystoids pattern characterized from segmental distribution of the radiolucent clusters along the bowel wall and cysts with a variable diameter between 5 and 30 mm and margins clearly identifiable [34] but with an uncommon small bowel localization.

CT is the most appropriate tool to localize the PI and to make differential diagnosis between primary PI and secondary PI basing on the shape of lesions.

The secondary forms usually have a different feature than idiopathic, showing a linear radiolucency within the wall of the gastrointestinal tract, parallel to the intraluminal gas. Sometimes the aspect can be fine bubble or mist [34].

Currently, there is no consensus on the appropriate management of PI. Since PI can represent a wide range of pathology, it is in itself not diagnostic of any certain condition. The variety of presentations of PI highlights the fact that clinicians should interpret radiographic findings in concert with the current clinical scenario in order to ensure a correct diagnosis and to guide one toward a suitable management. Around 50% of patients with PI can be successfully managed nonoperatively [35].

The idiopathic PI does not require surgical intervention in itself since some lesions disappear spontaneously within months or years [36], unless the breaking of bubbles occurs followed by periodic inflammatory episodes and adhesions with subobstruction as in our case.

Identification of cases in which laparotomy can be avoided, or deferred, is important in order to prevent unnecessary surgery with its associated morbidity and financial costs.

In conclusion the authors emphasize the role of TC in the localization of lesions and identification of PI pattern; the meticulous integration of the appearance on abdominal CT scan, the laboratory data, and clinical presentations permit clinicians to suspect the onset of complications and to distinguish benign from life-threatening PI and to decide whether or not urgent surgical intervention is necessary.
